# The moderating effect of marital conflict on the relationship between social avoidance and socio-emotional functioning among young children in suburban China

**DOI:** 10.3389/fpsyg.2022.1009528

**Published:** 2022-12-07

**Authors:** Jingjing Zhu, Mowei Liu, Xin Shu, Shuhui Xiang, Yaoqin Jiang, Yan Li

**Affiliations:** ^1^Early Childhood Education College, Shanghai Normal University, Shanghai, China; ^2^Trent University, Peterborough, ON, Canada; ^3^Shanghai Punan Kindergarten, Shanghai, China

**Keywords:** social avoidance, marital conflict, social adjustment, suburban China, preschoolers

## Abstract

Social avoidance has been found to be related to negative social adjustment, yet evidence of the relationship between social avoidance and social adjustment is very limited in suburban preschoolers in China. Moreover, the underlying mechanisms that help to explain the associations between social avoidance and socio-emotional adjustment remain poorly considered. The goal of the present study was to examine the moderating effect of marital conflict in the link between social avoidance and indices of socio-emotional functioning among young children in mainland China. Participants were *N =* 256 children aged from 49 to 72 months (125 boys, 131 girls, *M_age_* = 59.06 months, *SD* = 6.58) attending preschools/kindergartens in suburban areas of Shanghai, People’s Republic of China. Multi-source assessments were employed, with mothers reporting marital conflict as well as rating their children’s social withdrawal (i.e., social avoidance, shyness, unsociability), and teachers assessing indices of children’s socio-emotional functioning at school (i.e., anxious-fearful behavior, peer exclusion, and loneliness). Children were asked about their loneliness. Among the results, social avoidance was positively associated with anxious-fearful behavior, peer exclusion, and loneliness (marginal significance). Moreover, as hypothesized, marital conflict was found to exacerbate the relations between social avoidance and peer exclusion, and loneliness. Results are discussed in terms of the etiology and implications of social avoidance among young children in mainland China.

## Introduction

Peer interactions play a critical role in young children’s socio-emotional development because they facilitate the acquisition of positive social skills (Coplan and Ooi, 2014). However, there are some children who tend to remove themselves from opportunities and cannot learn from social stimuli. These children are described as being socially withdrawn (Rubin et al., 2009). Contemporary theoretical perspectives characterize social withdrawal as a multi-dimensional construct, based on motivational and emotional substrates. There are three subtypes of social withdrawal, namely shyness, unsociability, and social avoidance (Asendorpf, 1990; Rubin et al., 2009; Coplan et al., 2015; Sette et al., 2022). Shy children may engage in less social interactions due to fear and anxiety even though they desire peer interaction, whereas unsociable children may not be interested in but are receptive to social play. In contrast, social avoidance, the subtype that is of particular interest in the current study, refers to the combination of wanting to be alone and actively avoiding social settings. It has been argued that social avoidance can be an early indication of depression because socially avoidant children cannot derive pleasure from social exchanges (Coplan et al., 2018; Ding et al., 2019). Consistently, a recent study suggests that, among Italian primary school children, social avoidance is significantly associated with self-reported depression, particularly in older children (Sette et al., 2022). Previous studies indicated that social avoidance was associated with a host of adjustment difficulties, such as peer problems (e.g., rejection, exclusion, and victimization) and internalizing problems (e.g., loneliness, depression, and social anxiety; Bowker et al., 2017; Coplan et al., 2018; Eggum et al., 2022; Sette et al., 2022).

Most research on social avoidance and its developmental significance has been conducted in individualistic Western societies (Oyserman et al., 2002), and there has been very limited studies of social avoidance in collectivistic societies such as China. As the implications of social avoidance may differ across cultures, social avoidance might be viewed more negatively given the cultural emphasis on social connections and group harmony (Chen et al., 2011). The first goal of the present study was to examine the relationship between social avoidance and socio-emotional functioning among young children in China. Although social avoidance can be maladaptive in China, some factors may exacerbate or buffer children from social difficulties. It is important to examine potential factors that moderate the link between social avoidance and socio-emotional functioning. In the present study, we examined marital conflict as a potential moderator. Family is one of the most important influences in a child’s life. According to the family system approach, the family is composed of a variety of subsystems that involve multiple dyadic relationships such as marital relationships, sibling relationships, and parent–child relationships. Each subsystem influences every other subsystem in the family. High level of marital conflict affects child adjustment adversely (Hosokawa and Katsura, 2017; King and Mrug, 2018; Philbrook et al., 2018; Camisasca et al., 2019; Jarvis et al., 2020). Thus, more specifically, the second goal of the present study was to examine the potential moderating effect of marital conflict on the links between social avoidance and socio-emotional adjustment in a sample of kindergartners living in China.

## Overview of social avoidance in childhood

Children withdraw from social settings for different motivations (Coplan et al., 2015). For example, shyness is the result of an approach-avoidance motivational conflict (Asendorpf, 1990). *Shy* children withdraw because they are anxious about social contact and evaluations, despite the desire to interact with peers. A numerous of studies have indicated that, shyness is associated with adjustment difficulties such as internalizing problems (e.g., anxiety, loneliness), and negative peer experiences (e.g., exclusion) at various developmental stages (Bowker et al., 2012; Coplan et al., 2018; Poole et al., 2020; Sette et al., 2022). By contrast, *unsociable* children prefer to spend time alone, however, are able to function well in social settings (Coplan et al., 2018, 2021). In Western culture, unsociability is relatively benign, particularly in early childhood, and has not been associated with negative adjustment such as peer liking, peer disliking, exclusion, aggression, or victimization (Sette et al., 2017; Coplan et al., 2018; Eggum et al., 2022).

While of particular interest in the present study was *social avoidance,* which reflects a combination of high social avoidance and low social approach motivations (Asendorpf, 1990). *Socially avoidant* children tend to actively avoid social interactions. Social avoidance is considered an extreme form of shyness/social anxiety and represents an early manifestation of depression (Asendorpf, 1990; Schmidt and Fox, 1999). Consistent with this assertion, results from several studies found social avoidance is associated with maladjustment outcomes, such as peer exclusion, loneliness, and depression in Western societies (Bowker et al., 2017; Coplan et al., 2018, 2021; Eggum et al., 2022; Sette et al., 2022). For example, Sette et al. (2022) found that, after controlling for shyness and unsociability, social avoidance was positively associated with loneliness, regardless of children’s age or gender, in Italian children. Eggum et al. (2022) found that United States school-aged children, who were identified as having a social avoidance motivation by peers, were more likely to be disliked by their peers. Coplan et al. (2018) found that social avoidance was associated with peer difficulties among Canadian preschoolers after controlling for shyness and unsociability. Bowker and colleagues also found that social avoidance was related to peer exclusion and loneliness after controlling for shyness and unsociability in Indian adolescent (Bowker and Raja, 2011; Bowker et al., 2012). Thus, the existing literature generally points toward a similar unique pattern of risk for maladjustment associated with social avoidance.

### Social avoidance in China

According to the literature review, the exploration of the effects of social avoidance has been largely conducted in Western samples, and there has been a paucity of research about social avoidance in collectivistic societies such as China. According to Ran et al. (2022), to date, only 5 out of 43 empirical articles have examined the relationship between children’s social avoidance and peer problems, the rest studies either focus on shyness, unsociability or adult participants. Thus, the present study would fill this gap. In Chinese societies, where personal interdependence and group affiliation was highly endorsed, avoiding peer contact and removing oneself from group activities is viewed as selfish (Chen et al., 2019). As a result, shy and unsociable children were found to be exposed to negative peer experiences, internalizing problems, and poorer academic achievement (Liu et al., 2015, 2016; Ding et al., 2022; Zhu et al., 2022). Although there is limited empirical evidence of the developmental significance of social avoidance in China, existing literature suggests that social avoidance, compared with shyness and unsociability, is associated with higher level of maladjustment outcomes, given the cultural emphasis on group harmony and interdependence (Ding et al., 2015, 2019, 2022; Sang et al., 2018). For instance, Coplan et al. (2016) found for 10–12-year-old children, compared to shy and unsociable children, socially avoidant children reported higher internalizing problems (i.e., depression, social anxiety). Sang et al. (2018) also reported that social avoidance was associated with teacher-rated dysregulation and self-reported internalizing problems among Chinese early adolescents. Xu et al. (2022) found that individuals (aged from 19 to 64) with high social avoidance in China reported the lowest level of well-being than those from other subgroups during the COVID-19 pandemic. Of note, these studies were conducted with samples of older children, adolescents, and adults.

Little is known about the relationship between social avoidance and social adjustment, and its potential mechanism among young Chinese children. Our review of the literature only uncovered three previous studies pertaining to social avoidance among young children in China. The findings of these studies are, nonetheless, inconsistent. Ding et al. (2015) presented SK and first graders with peer scenarios that demonstrated shy, unsociable, socially avoidant, and socially competent behaviors. It was found that young children were able to distinguish these different behaviors and associate the most negative developmental outcomes with socially avoidant peers. In contrast, Zhu et al. (2020a, 2021) examined the relationship between social avoidance and social functioning in preschoolers. It was found that social avoidance was not significantly associated with social adjustment difficulties such as peer exclusion and anxiety. Given the lack of previous studies on social avoidance in early childhood, one goal of the current study was to explore the implications of social avoidance in a sample of Chinese preschoolers.

### Role of marital conflict

Marital conflict refers to high levels of disagreement between spouses characterized by hostility, anger, and tension in the relationship (Buehler et al., 1998). The effect of marital conflict on child development can be direct or indirect. As a direct source of influence, parental conflict may increase negative emotions, emotional insecurity, threat appraisal, self-blaming attribution, and internalizing and externalizing problems in children at different ages (Davies et al., 2018; Philbrook et al., 2018; El-Sheikh et al., 2019; Fosco and Lydon-Staley, 2019; Hibel and Mercado, 2019; Adare et al., 2021; Craft et al., 2021; de Silva Aryanne et al., 2021; Xu et al., 2021; Lee et al., 2022; MacNeill and Fosco, 2022; Sloan et al., 2022). For example, as young as 5–8 months of age, infants were found to display increased negative emotions, as measured by cortisol reactivity, after exposure to parental conflict (Hibel and Mercado, 2019). Kindergarteners who experienced parental conflict displayed a higher level of emotional insecurity in grade 2, which led to problem behaviors in grade 7 (Cummings et al., 2012). Experiencing parental conflict may also affect cognitive appraisal in children and adolescents. Children may interpret relationships as a threat and/or blame themselves for parental conflict, and these attributions may affect individual development and adjustment (Grych and Fincham, 1993; Fosco and Lydon-Staley, 2019; Xu et al., 2021). Witnessing parental conflict can also affect the autonomic nervous system (ANS) functioning and lead to internalizing and externalizing problems in adolescents (Philbrook et al., 2018). The development of externalizing problems in children who are exposed to marital conflict is also explained by Social Learning Theory (Bandura, 1977) which posited that children may learn aggressive conflict resolution during marital conflict, which leads to negative adjustment outcomes.

As an indirect source of influence, marital conflict may disrupt parental behaviors and parent–child relationships, which in turn affect child adjustment. According to the family system approach, parent–child relationships can be affected by other dyadic relationships within the family (Cox and Paley, 2003). The Spill-Over Hypothesis suggested that negative emotions in conflictual marital relations can “overflow” into parent–child relationships (Erel and Burman, 1995). Parents who experience spousal conflict are more likely to become emotionally disengaged and insensitive, use punitive, excessive control strategies, and display low levels of parental acceptance (McCoy et al., 2013; Tavassolie et al., 2016; Hibel and Mercado, 2019; Cross et al., 2021; McRae et al., 2021; Hess, 2022). These inadequate parental behaviors result in negative developmental outcomes in children (Coln et al., 2013; Xiao et al., 2021). Thus, marital conflict is increasingly becoming an important factor in the study of child psychological development, and the role of marital conflict should be considered particularly when the link between social avoidance and social adjustment in children is concerned. According to the *stress-diathesis* model (Monroe and Simons, 1991), environmental stressors may be more influential to some individuals depending on diathesis or vulnerability factors, such as their behavioral, physiological, or genetic characteristics. Children who are socially avoidant are at risk for developmental adversity due to their high level of social anxiety and avoidant tendencies. For these children, participation in social activities may require safety assurance, positive appraisal of the relationship, and adequate environmental support, such as parental warmth and sensitivity. These requirements are challenging when children are exposed to a high level of parental conflict.

As discussed earlier, parental conflict may affect individual’s adjustment directly or indirectly. According to Emotional Security Theory (Davies and Cummings, 1994), feelings of security within family are important for child adjustment. Children who are socially avoidant are extremely anxious about social interactions. The emotional distress and physiological arousal may be heightened when emotional security within family is not well developed due to exposure to marital conflict. Moreover, the cognitive-contextual framework (Grych and Fincham, 1993) suggests that children can develop expectations of danger and conflict in their social interactions based on their experiences of parenting conflict at home. For example, Sloan et al. (2022) found that when children exposed to intense, frequent, and unresolved parental conflict in the past, they were more likely to become sensitive to conflict and exhibit more negative responses and threat appraisal toward daily parental conflict. The threat appraisal of interpersonal relationship that children have may refrain themselves from social activities, which could lead to more social adjustment problems.

In addition, parents who experience spousal conflict are likely to adopt negative and ineffective parenting practices, which may fail to provide the support and sensitive responses for anxious, socially avoidant children. For instance, it was found that parents in marital conflict were more likely to become emotionally unavailable, insensitive, and psychologically controlling (Sturge-Apple et al., 2006; Davies et al., 2009; Koçak et al., 2017; Xiao et al., 2021; Hess, 2022). Zhu et al. (2020b) found maternal psychological control moderated the links between social avoidance and social adjustment among Chinese preschoolers. At higher levels of maternal psychological control, social avoidance was associated with negative adjustment outcomes, whereas at lower levels of maternal psychological control there was no association.

To summarize, the association between social avoidance and children’s social adjustment may be moderated by marital conflict. For children who are socially avoidant, exposure to marital conflict can be detrimental. The way in which parents interact with each other is the one, if not the only, dyadic family relationship that a child witnesses as a third party every day. In the present study, we sought to examine the moderating role of marital conflict on the relationship between social avoidance and social adjustment among Chinese preschoolers (Sette et al., 2018).

### The present study

Because shyness, unsociability, and social avoidance are related but distinctive constructs, it is necessary to control for any shared variance to explore the unique effects and implications of social avoidance on socio-emotional functioning (Coplan et al., 2018; Sette et al., 2022). There are only limited studies that have explored the implications of social avoidance in young Chinese children (Zhu et al., 2020b, 2021). Moreover, despite a number of research suggesting that both social avoidance and marital conflict play very important roles in affecting Chinese children’s social adjustment (Xuan et al., 2018; Xiao et al., 2021; Zhu et al., 2021), to date nearly no study has examined the interactions of social avoidance and marital conflict on Chinese preschoolers’ social adjustment. Accordingly, the research questions for the present study were: (1) What is the unique relationship between social avoidance and socio-emotional functioning; and (2) Does the marital conflict play a moderating role on the associations between social avoidance and indices of socio-emotional functioning among preschool children living in suburban China.

While previous studies focused on urban areas, we chose the suburban context in this study because it has been largely overlooked in past research. Chinese culture varies by geographic areas. The urban area is complicated by Western ideologies, which encourages values such as independence, competitiveness, and assertiveness, whereas the traditional Chinese values such as modesty, interpersonal connectedness are highly endorsed in rural areas (Chen, 2015). Families in suburban areas may hold diverse values and lifestyles (e.g., Mao, 2009). Also, the limited number of studies about social avoidance among Chinese young children have been conducted on preschool children in an urban context (Ding et al., 2015; Zhu et al., 2020b, 2021). The social adjustment of children from suburban areas remains unclear. Therefore, we sought to fill in the gaps of previous research by exploring the effects of social avoidance on socio-emotional adjustment among preschool children living in a suburban area in China.

**Figure 1:**
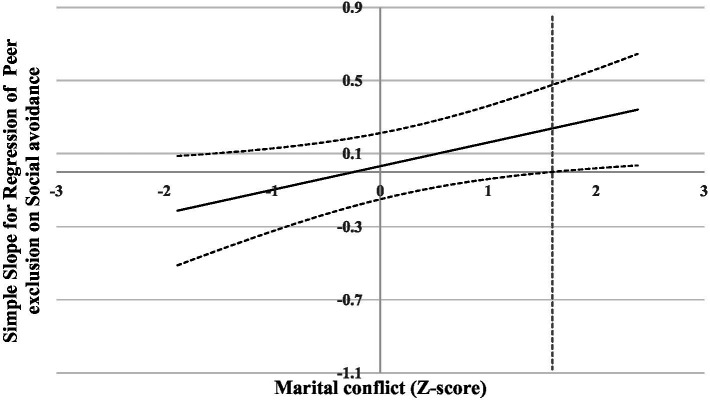
Johnson-Neyman regions of significance and confidence bands for social avoidance along marital conflict in relation to peer exclusion. Solid diagonal line represents the regression coefficient for social avoidance along marital conflict. Dashed diagonal lines are confidence bands—upper and lower bounds of 95% confidence interval for social avoidance coefficient along marital conflict. The dashed vertical red line indicates the point along marital conflict at which the social avoidance regression coefficient transitions from non-significance (left of dashed vertical line) to statistical significance (right of dashed vertical line). The value of the dashed vertical line is 1.60.

Most of the previous studies reported that social avoidance was associated with negative adjustment among older Chinese children, adolescents, and adults (Coplan et al., 2016; Sang et al., 2018; Ding et al., 2022; Xu et al., 2022), The only three studies involving young Chinese children in urban areas yielded mixed results. Compared with hypothetical shy and unsociable peers, hypothetical socially avoidant peers were more negatively viewed by young children (Ding et al., 2015). However, studies examining “real” children suggested a lack of unique associations between social avoidance and adjustment difficulties (Zhu et al., 2020b, 2021). Because cultural traditional values such as connectedness and interdependence are more emphasized in suburban areas than in urban areas, deliberate social interaction avoidance may be viewed more negatively. Therefore, in the present study, we hypothesized that social avoidance would be uniquely and positively associated with indices of socio-emotional problems including anxious-fearful behavior, peer exclusion, and loneliness. Moreover, previous studies have examined the interactions of children’s characteristics and marital conflict in predicting children’s social adjustment (David and Murphy, 2007; Hentges et al., 2015; Thompson et al., 2020; Xiao et al., 2021; Xu et al., 2021). The central goal of the present study was to further examine the interaction effects of social avoidance and marital conflict on children’s socio-emotional functioning. We hypothesized that marital conflict would play a moderating role in the relationship between social avoidance and adjustment difficulties. Specifically, we expected that higher levels of marital conflict would exacerbate socially avoidant children’s risk for anxious-fearful, peer exclusion, and loneliness whereas lower levels of marital conflict would mitigate these associations (see Figure 1).

## Materials and methods

### Participants

We randomly selected 256 children from two public kindergartens from suburban areas in Shanghai, China (125 boys, 131 girls, *M_age_* = 59.06 months, *SD* = 6.58). All children were of Han ethnicity, which is the predominant ethnic group in China (nearly 97% of the population). Maternal and paternal scores were averaged to construct a broader measure of parental education (with higher scores representing higher education). The demographic data for the sample were similar to those previously reported concerning the suburban population in Shanghai (Bureau of Statistics of China, 2010). All participating teachers were female. Teachers were of varied ages from 21 to 40 years old, and had a wide range of teaching experience from 2 years or less to 20 years (Table 1).

**Table 1:** Demographic statistics.

Variables	Female	Male	Total
**Children**			
Grade: *n* (%)			
Junior class	85	74	159 (62%)
Senior class	46	51	97 (38%)
Total	131 (51%)	125 (49%)	256 (100%)
Age (month): M (SD)	58.27 (6.82)	59.90 (6.22)	59.06 (6.58)
**Parents**			
Education level: *n* (%)			
High school	33 (13%)	39 (15%)	71 (14%)
Junior college degree	69 (27%)	51 (20%)	120 (23%)
Bachelor’s degree	123 (48%)	125 (49%)	248 (49%)
Postgraduate degree	31 (12%)	41 (16%)	72 (14%)
Total	256 (100%)	256 (100%)	512 (100%)

### Procedure

Mothers reported their children’s social avoidance and marital conflict. Information on children’s socio-emotional functioning was obtained from teacher-ratings. Children were asked about their loneliness one-on-one in their kindergarten classrooms. All measures in the present used have proven to be reliable, and appropriate among young Chinese children (e.g., Zhang et al., 2009; Zhu et al., 2018, 2019; Zhu et al., 2021). The present study was reviewed and approved by the ethics review board of BLINDED FOR PEER REVIEW. Written consent was obtained from the parents of all the children through the school. The participation rate was 98%. Data were collected in 2019.

### Measures

*Social avoidance.* Mothers completed the Chinese version of *Child Social Preference Scale-3* (CSPS-3; Coplan et al., 2018; Zhu et al., 2021). Of particular interest was the subscale assessing social avoidance (4 items, e.g., “My child often goes out of his/her way not to play with other children,” Cronbach’s α = 0.77). Given the shared conceptual overlap and similar implications in Chinese youth and urban preschoolers, it is essential to control for any shared variance with shyness and unsociability when exploring the implications of social avoidance among Chinese children (e.g., Sang et al., 2018; Zhu et al., 2020a). In this regard, mother also rated shyness (7 items, e.g., “My child will turn down social initiations from other children because he/she is “shy” Cronbach’s α = 0.88), and unsociability (4 items, e.g., “My child often seems content to play alone,” Cronbach’s α = 0.72). All items were rated on a five-point scale from 1 (not at all) to 5 (a lot). Higher scores for CSPS-3 subscales indicated higher levels of social avoidance, shyness, and unsociability, respectively. All 15 items were treated as ordinal variables and specified to load on one latent factor. The CFA model yielded a good fit (comparative fit index [CFI] = 0.99, root mean square error of approximation [RMSEA] = 0.07), with factor loadings of the items ranging from 0.64 to 0.88. McDonald’s Omega was calculated based on the factor loadings as a more accurate and unbiased estimate of internal reliability (Dunn et al., 2014; Peters, 2014). The McDonald’s omega was 0.78, 0.89 to 0.72 for social avoidance, shyness, and unsociability, separately, and the composited reliability was ranging from 0.81 to 0.92, indicating good reliability. The average variance was 0.67, 0.63, and 0.52 for social avoidance, shyness, and unsociability, separately, thus the convergent validity was acceptable. The measure has been shown to be reliable and valid in young children in China (Zhu et al., 2021).

#### Marital conflict

Mothers completed the Chinese version of *Dyadic Adjustment Scale* (DAS; Spanier, 1976; Zhang et al., 2009). Of particular interest was the subscale of dyadic conflict (8 items, e.g., “Do you and your partner have conflicts and arguments over handling family finance,” Cronbach’s α = 0.80), scored on a 4-point Likert scale (from 1 = “never” to 4 = “always agree”), with higher scores indicating more marital conflict. All 8 items were treated as ordinal variables and specified to load on one latent factor. The CFA model yielded a good fit (comparative fit index [CFI] = 1.00, root mean square error of approximation [RMSEA] = 0.04), with factor loadings of the items ranging from 0.42 to 0.82. The McDonald’s omega was 0.81 and the composite reliability was 0.86, indicating good reliability. The average variance extracted (AVE) was 0.44. Previous studies showed AVE should exceed 0.5 under ideal conditions, but 0.36 ~ 0.50 are acceptable (Fornell and Larcker, 1981), thus the convergent validity was acceptable. The measure has been shown to be reliable and valid in Chinese culture context (Zhang et al., 2009).

#### Teacher ratings of problems

Teachers completed the Chinese version of *Child Behavior Scale* (CBS; Ladd and Profilet, 1996; Zhu et al., 2018). The present study used the subscales of anxious-fearful behavior (4 items, e.g., “Is worried,” Cronbach’s α = 0.73), and peer exclusion (7 items, e.g., “Excluded from peer’s activities,” Cronbach’s α = 0.89). Items were rated on a three-point scale (from 1 “does not apply” to 3 “certainly applies”). For anxious-fearful behavior (comparative fit index [CFI] = 1.00, root mean square error of approximation [RMSEA] = 0.09), with factor loadings of the items ranging from 0.71 to 0.92 in the CFA model. The McDonald’s omega was 0.73, and composite reliability was 0.97, indicating good reliability. The average variance was 0.82, thus the convergent validity was acceptable. For peer exclusion (comparative fit index [CFI] = 1.00, root mean square error of approximation [RMSEA] = 0.04), with factor loadings of the items ranging from 0.84 to 0.95 in the CFA model. The McDonald’s omega was 0.91, and composite reliability was 0.88, indicating good reliability. The average variance was 0.64, thus the convergent validity was acceptable. The measure has been shown to be reliable and valid in young children in China (Zhu et al., 2018).

#### Loneliness

The Chinese version of *Loneliness Questionnaire for Young Children* (LSDQ, Cassidy and Asher, 1992; Zhu et al., 2019) was used to assess children’s loneliness, which includes 24 items rated on a 3-point Likert scale, 16 of which assess loneliness (e.g., “Are there kids you can go to when you need help in school?,” Cronbach’s a = 0.81), and the remaining 8 items about children’s hobbies and other activities, designed to help children relax during the process. Items are coded 1 (yes), 2 (sometimes), or 3 (no). All 16 items were added up to create a loneliness score. Higher scores reflect a higher level of loneliness. All 16 items were treated as ordinal variables and specified to load on one latent factor. The CFA model yielded a good fit (comparative fit index [CFI] = 1.00, root mean square error of approximation [RMSEA] = 0.03), with factor loadings of the items ranging from 0.33 to 0.79 in the CFA model. The McDonald’s omega was 0.81, and composite reliability was 0.88, indicating good reliability. The average variance was 0.33, thus the convergent validity was acceptable. The measure has been shown to be reliable and valid in young children in China (Zhu et al., 2019).

### Analytical strategy

Data analysis was performed using IBM SPSS for Windows, version 22. First, we used *t*-test to examine gender differences on all the studied variables, to decide which variables should be controlled in the further analysis and used Pearson Correlation to examine the inter-correlations between studied variables. Then, the present study adopted Hayes’s PROCESS macro model 1 with non-parametric bootstrapping with 5,000 resamples (Hayes, 2013) to probe the moderating effect of marital conflict on the link between social avoidance and indices of socio-emotional adjustment. The moderating effect was thought to be significant when the zero was not included in 95% bias-corrected confidence interval (CI) of an interaction term (social avoidance × marital conflict; Preacher and Hayes, 2008). Finally, the Johnson-Neyman (J-N) Technique (Johnson and Fay, 1950) was employed to probe those significant interactions, which allowed us to estimate a region of significance for the simple slope of a predictor conditioned on the value of the continuous moderator.

## Results

### Preliminary analyses

Results from *t*-tests indicated that teachers reported significantly more peer exclusion for boys than girls (*M_boy_* = 1.20, *SD* = 0.36; *M_girl_* = 1.08, *SD* = 0.21, *t* = 3.29, *p* = 0.001, Cohen’s *d* = 0.41), and significantly higher child-report loneliness for boys than girls (*M_boy_* = 0.59, *SD* = 0.31; *M_girl_* = 0.51, *SD* = 0.27, *t* = 2.21, *p* = 0.03, Cohen’s *d* = 0.28). Gender had no effect on other variables.

Correlations between all studied variables are displayed in Table 2. Results were largely in keeping with conceptual expectations, which indicated that social avoidance, shyness, and unsociability were all significantly and positively associated with each other moderately, reflecting shared variance among different substrates of social withdrawal. Social avoidance was positively correlated with anxious-fearful behavior, peer exclusion, and loneliness (marginal significance). Marital conflict was positively associated with shyness (marginal significance), unsociability, and social avoidance, while not associated with any of the adjustment variables. Also indicates that parent education was only correlated with unsociability, but not with other variables, while not to be controlled.

**Table 2:** Inter-correlations for all study variables (*N* = 256).

	1	2	3	4	5	6	7	8	9
1. Parent education	-								
2. Child age	−0.0.03	-							
3. Shyness	0.12	−0.07	-						
4. Unsociability	0.13^*^	−0.09	0.65^***^	-					
5. Social avoidance	0.09	−0.1	0.64^***^	0.73^***^	-				
6. Marital conflict	−0.04	0.05	0.11^+^	0.12^*^	0.17^**^	-			
7. Anxious-fearful	0.08	−0.06	0.17^**^	0.19^**^	0.17^**^	0.08	-		
8. Peer exclusion	−0.03	0.05	0.18^**^	0.26^***^	0.22^**^	0.03	0.39^***^	-	
9. Loneliness	−0.07	0.05	0.07	0.09	0.10^+^	−0.08	0.09	0.26^***^	-
*M*	-	59.06	1.93	1.79	1.4	2.94	1.22	1.14	0.55
*SD*	-	6.58	0.71	0.61	0.5	0.5	0.55	0.3	0.29

* *p* < 0.05;

** *p* < 0.01;

*** *p* < 0.001;

^+^*p* < 0.10.

### Social avoidance, marital conflict, and socio-emotional functioning

The goal of the following analyses was to explore the potential moderating role of marital conflict in the relations between social avoidance and indices of socio-emotional difficulties (i.e., anxious-fearful behavior, peer exclusion, and loneliness) while controlling for parent education, child age, gender, shyness, and unsociability. We used Hayes’s (2013) PROCESS macro (Model 1) to examine the moderating effects of marital conflict. All continuous studied variables were standardized (Aiken et al., 1991). The results indicated that the significant interactions between social avoidance and marital conflict in relation to peer exclusion, and loneliness, while there is no significant interaction in relation to anxious-fearful behavior (see Table 3).

**Table 3:** Effects of social avoidance, marital conflict (controlling for gender) in relation to indices of social adjustment.

Social adjustment variables							
Predictor	*B*	*SE*	*t*-Value	95% CI	R^2^	Δ*R*^2^	Δ*F*
**Anxious-fearful**							
Gender	0.02	0.13	0.15	[−0.23, 0.26]	0.05	0	0.58
Shyness	0.05	0.09	0.58	[−0.12, 0.22]			
Unsociability	0.13	0.1	1.32	[−0.06, 0.32]			
Social Avoidance	0.05	0.1	0.53	[−0.14, 0.24]			
Marital Conflict	0.05	0.06	0.84	[−0.07, 0.18]			
Avoidance × Marital Conflict	−0.05	0.06	−0.76	[−0.17, 0.07]			
**Peer exclusion**							
Gender	−0.4	0.12	−3.38^***^	[−0.64, −0.17]	0.13	0.02	5.01
Shyness	0.05	0.08	0.69	[−0.11, 0.22]			
Unsociability	0.16	0.09	1.75	[−0.02, 0.35]			
Social Avoidance	0.03	0.09	1.24	[−0.07, 0.30]			
Marital Conflict	−0.03	0.09	0.34	[−0.15, 0.21]			
Avoidance × Marital Conflict	0.13	0.06	2.24^*^	[0.02, 0.24]			
**Loneliness**							
Gender	−0.29	0.09	−2.38^*^	[−0.54, −0.05]	0.07	0.02	6.19
Shyness	0.04	0.09	0.52	[−0.13, 0.21]			
Unsociability	−0.02	0.l0	−0.19	[−0.21, 0.17]			
Social Avoidance	0.07	0.1	0.69	[−0.12, 0.25]			
Marital Conflict	−0.13	0.06	−2.05^*^	[−0.25, −0.00]			
Avoidance × Marital Conflict	0.15	0.06	2.49^*^	[0.03, 0.27]			

CI, confidence interval.

* *p* < 0.05;

** *p* < 0 0.01;

*** *p* < 0.001.

To probe significant interaction terms, simple slope tests were first conducted, followed by the application of the Johnson and Fay (1950)’s Johnson–Neyman (J-N) technique to estimate regions of significance (standardized scores were used for all the predictors). This J-N technique estimated the region of significance for the predictor’s (i.e., social avoidance) simple slope conditioned on the value of the continuous moderator variable (i.e., marital conflict).

For the prediction of anxious-fearful behavior, there was no significant main effect of social avoidance and marital conflict, nor the significant interactions of social avoidance and marital conflict.

For the prediction of *peer exclusion*, there was no significant main effect of social avoidance and marital conflict. However, a significant interaction of social avoidance and marital conflict was found. Results of the follow-up analysis (see Figure 1) indicated that, when the level of marital conflict was lower than 1.60, social avoidance was not associated with peer exclusion. However, when the level of marital conflict was higher than 1.60, social avoidance was significantly and positively associated with peer exclusion. Thus, higher levels of marital conflict appeared to exacerbate the positive association between social avoidance and peer exclusion.

**Figure 2:**
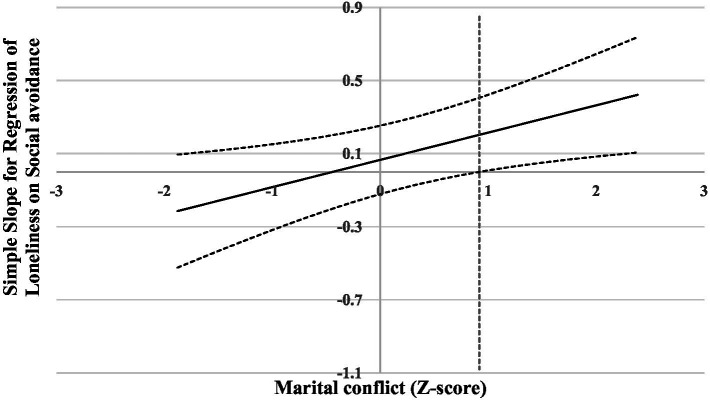
Johnson-Neyman regions of significance and confidence bands for social avoidance along marital conflict in relation to loneliness. Solid diagonal line represents the regression coefficient for social avoidance along marital conflict. Dashed diagonal lines are confidence bands—upper and lower bounds of 95% confidence interval for social avoidance coefficient along marital conflict. The dashed vertical red line indicates the point along marital conflict at which the social avoidance regression coefficient transitions from non-significance (left of dashed vertical line) to statistical significance (right of dashed vertical line). The value of the dashed vertical line is 0.92.

For the prediction of *loneliness*, a significant main effect of marital conflict was found. This main effect was superseded by a significant interaction of social avoidance and marital conflict. Results of the follow-up analysis (see Figure 2) indicated that, when the level of marital conflict was lower than 0.92, social avoidance was not associated with loneliness. However, when the level of marital conflict was higher than 0.92, social avoidance was significantly and positively associated with loneliness. Thus, higher levels of marital conflict appeared to exacerbate the positive association between social avoidance and loneliness.

## Discussion

The “functioning” of social behavior is determined by social and cultural contexts (Chen and French, 2008). For social withdrawal, especially shyness and unsociability, previous studies mainly focused on Western and urban Chinese children (Liu et al., 2015; Coplan et al., 2018; Ding et al., 2019; Poole et al., 2020; Eggum et al., 2022; Sette et al., 2022). To expand the research in this area, the present study examined the relations between social avoidance and socio-emotional functioning, and further explored the moderating role of marital conflict in the links between social avoidance and indices of socio-emotional functioning among young children in the context of contemporary suburban China.

### Implications of social avoidance in China

The first purpose of the present study was to examine the unique relationship between social avoidance and socio-emotional functioning in young children. It was expected that, after controlling for shyness and unsociability, social avoidance would be positively associated with socio-emotional problems. This hypothesis was not supported. Our findings indicated that, overall, social avoidance was significantly associated with the widest range of socio-emotional difficulties, including anxious-fearful behavior, peer exclusion, as well as loneliness in suburban Chinese young children. However, after controlling for shyness and unsociability using Hayes’s (2013) PROCESS macro (Model 1), social avoidance was no longer significantly predictive of those adjustment problems, which was consistent with the findings of their urban counterparts (Zhu et al., 2021). Although in suburban areas where traditional values such as group affiliation and interpersonal independence are still highly valued, social avoidance was not associated with negative outcomes after controlling for shyness and unsociability. The lack of unique relationship between social avoidance and socio-emotional functioning in the present study might be due to the behavioral characteristics of children at young age. It has been argued that preschoolers tend to be self-centered and commonly engage in solitary play (Coplan et al., 2004). The anxious nature of social avoidance can be overshadowed by its solitary behavioral manifestation. Consequently, social avoidance may not be related to adjustment difficulties during kindergarten. However, the current findings were not in keeping with previous studies conducted in Western cultures (Bowker and Raja, 2011; Coplan et al., 2018; Clifford et al., 2021) and Chinese older children (Coplan et al., 2016; Sang et al., 2018; Ding et al., 2022), in their research, social avoidance was associated with more negative outcomes after controlling for shyness and unsociability. Given that the current study is the first to explore the links between social avoidance and adjustment in suburban young Chinese children, further research is warranted.

### Role of marital conflict

Although after controlling for shyness and unsociability, social avoidance was not associated with social adjustment, it is possible that the links between social avoidance and indices of socio-emotional functioning are moderated by marital conflict. The second purpose of the present study was to explored the role of marital conflict in the development and implications of social avoidance among suburban young children. It was hypothesized that higher levels of marital conflict would exacerbate associations between social avoidance and indices of socio-emotional difficulties, including anxious-fearful behaviors, peer exclusion, and loneliness among suburban young Chinese children while controlling for shyness and unsociability. Our hypothesis was largely supported. Marital conflict was found to exacerbate the relationships between social avoidance and teacher-rated peer exclusion and child self-reported loneliness. However, the moderating effect was not found for the link between social avoidance and teacher-rated anxious-fearful behaviors.

According to Emotional Security Theory (Davies and Cummings, 1994; Davies and Martin, 2013), attaining and maintaining emotional security is a fundamental task for an individual. When emotional security is compromised by negative interpersonal relationships such as parental conflict, children take steps to de-escalate or avoid existing and potential threats to maintain their sense of security. The avoidance tendency can be even stronger for socially avoidant children who are motivated to manage/mask social anxiety by refraining from social interactions. Consistently, no significant interaction between social avoidance and marital conflict was found in predicting anxious-fearful behaviors. It is worth noting that, although socially avoidant children who are exposed to intensive parental conflict may not display significantly high level of teacher-rated anxious-fearful behaviors, anxiety and fear may be internalized and concealed. Longitudinal studies should be conducted in the future to examine the long-term relationships between social avoidance, marital conflict, and children’s socio-emotional functioning.

In addition, the effects of marital conflict on socially avoidant children’s anxious-fearful behavior may vary by the content of the conflict. Previous research suggests that children display higher levels of fear and worry when the content of marital conflict is child-related (Fincham and Osborne, 1993; Oh et al., 2011; Camisasca et al., 2019). Thus, Further exploration of the effects of different types of marital conflict on anxious-fearful behaviors in socially avoidant children is necessary in the future.

Consistent with our hypothesis, marital conflict was found to moderate the relationships between social avoidance and teacher-rated peer exclusion and child self-reported loneliness. There are several conceptual mechanisms that may underlie the exacerbating effect of marital conflict on the risk associated with social avoidance. According to the Cognitive Contextual Framework (Grych and Fincham, 1993), when experiencing marital conflict, children are likely to form threat appraisal and develop negative views of social relationship. To avoid the potential harms associated with social interactions, children may purposefully stay away from peers. Moreover, parents with higher levels of marital conflict may adopt inappropriate problem-solving behaviors, such as physical or verbal aggression. According to Social Learning Theory (Bandura, 1977), children may learn these interactive social skills through observational learning and apply them to peer context. Their lack of peer interactions and adaptive social skills may make them susceptible to peer exclusion. Our research finding suggests that, when socially avoidant children are in a family environment with high marital conflict, they experience higher levels of peer exclusion.

Significant moderating effect of marital conflict was also found when the relationship between social avoidance and loneliness was examined. It was found that socially avoidant children were more likely to express feelings of loneliness when they were exposed to intensive parental conflict. Loneliness refers to a state of mind characterized by negative emotion and discomfort due to physical isolation and/or the inconsistency between the expected and the reality in social relationships (De Jong Gierveld and Van Tilburg, 2010; Russell et al., 2012). Despite their active avoidance of social interactions, socially avoidant children, as all others, pursuit the fundamental and essential human need of emotional security (Andersen et al., 2000; Davies and Martin, 2013). However, their emotional security need may not be easily met due to their high avoidance and low approach motivations. Given that social avoidance is conceptualized as an indicator of childhood vulnerability to stress (Coplan et al., 2015), according to the *stress-diathesis* model (Monroe and Simons, 1991), this sensitivity to stress would be aggravated by repeated exposure to chronic stressors, including marital conflict (Hentges et al., 2015; Thompson et al., 2020). Previous studies reported that marital conflict can adversely affect child functioning *via* its negative impact on parenting. Marital conflict is related to maternal insensitivity, negative parent–child interactions, and harsh or neglectful parenting behaviors (Gustafsson et al., 2015; Hosokawa and Katsura, 2017; Smith et al., 2019; Xiao et al., 2021), which leads to children’s maladjustment. For socially avoidant children who are already prone to socio-emotional difficulties, a lack of warm and supportive parenting might be particularly damaging (Hastings et al., 2019). Consequently, socially avoidant children who are exposed to marital conflict may experience physical solitude (partly due to their active avoidance of social context) and emotional sadness and discomfort due to their unmet need for emotional security in social relationship. In addition, it was found in the current study that, when experiencing marital conflict, socially avoidant children are also likely to be excluded by their peers, which could be connected to their feelings of loneliness as well.

### Limitations and directions for future research

Results from the present study provided some of the first insights into the etiology and implications of social avoidance among young children in mainland China. Our findings added to the field of research on marital conflict interacting with the characteristics of the child in the prediction of child dysfunction (David and Murphy, 2007; Hentges et al., 2015; Thompson et al., 2020). Despite the contribution of this research to the existing literature, there are some limitations that should be noted. To begin, because the present study was a correlational design, we cannot preclude alternative interpretations of the relationship between social avoidance and socio-emotional difficulties. For example, it is possible that children who are experiencing peer exclusion may evoke worsened socially avoidant behavior over time (Bowker and Raja, 2011). A longitudinal design should be considered in future research to test these effects (MacKinnon, 2008).

Moreover, marital conflict was reported only by mothers. Future studies should also assess father-reported marital conflict and compare the difference between mother-reported and father-reported marital conflict on the relation between social avoidance and socio-emotional difficulties. Also, the present study only examined marital conflict and a specific subset of outcome variables. Future studies should also assess other aspects of family factors (e.g., parental emotional socialization, parent–child relationships) for both mothers and fathers, as well as a wider host of adjustment variables (e.g., learning problems, self-esteem).

The sample in the present study was recruited from suburban Shanghai. Future studies should also consider samples from lower SES, such as rural areas, where the effects of marital conflict appear to be particularly pronounced (Lei et al., 2019). Finally, future studies should corroborate longer-term risks for socio-emotional difficulties for socially avoidant young children in China, targeted prevention and intervention programs should be considered (Li et al., 2016).

Despite the limitations, the results of the present study contribute to our understanding of relations between social avoidance and adjustment outcomes and the role of marital conflict in contemporary suburban Chinese children. In addition, our findings may have practical implications for early intervention programs for socially avoidant children. For example, parents need to remember that marital conflict plays a risky role in children’s social adjustment. Thus, parents could adopt constructive strategies to decrease the level of their marital conflict, such as negotiation, helpfulness, and resourcefulness. Parents should create a safe emotional atmosphere for children, promote parent–child interactions, then enhance children’s self-esteem. Parents could also create opportunities for their children to participate in group activities such as sports to improve their interpersonal skills, experience positive emotions, and thus reducing their internalizing problems.

## Data availability statement

The data and materials used during the current study are available from the corresponding author YL, on reasonable request.

## Ethics statement

The studies involving human participants were reviewed and approved by Shanghai Normal University. Written informed consent to participate in this study was provided by the participants’ legal guardian/next of kin.

## Author contributions

JZ designed the study and wrote the manuscript. ML provided feedback on writing and editing of manuscript. XS assisted with data analyses and provided feedback on writing and editing of manuscript. SX collaborated with data analyses. YJ collaborated with data collection. YL involved in data design and collection of the large data set and provided feedback on writing. All authors contributed to the article and approved the submitted version.

## Funding

This work was supported by funding from the National Social Science Fund of China (21CSH048).

## Conflict of interest

The authors declare that the research was conducted in the absence of any commercial or financial relationships that could be construed as a potential conflict of interest.

## Publisher’s note

All claims expressed in this article are solely those of the authors and do not necessarily represent those of their affiliated organizations, or those of the publisher, the editors and the reviewers. Any product that may be evaluated in this article, or claim that may be made by its manufacturer, is not guaranteed or endorsed by the publisher.
